# Optimal Clinical Management and the Molecular Biology of Angiosarcomas

**DOI:** 10.3390/cancers12113321

**Published:** 2020-11-10

**Authors:** Tom Wei-Wu Chen, Jessica Burns, Robin L. Jones, Paul H. Huang

**Affiliations:** 1 Department of Oncology, National Taiwan University Hospital and Graduate Institute of Oncology, National Taiwan University College of Medicine, Taipei 100, Taiwan; 2 Division of Molecular Pathology, The Institute of Cancer Research, London SW3 6JB, UK; jess.burns@icr.ac.uk; 3 Sarcoma Unit, The Royal Marsden NHS Foundation Trust and Institute of Cancer Research, London SW3 6JJ, UK; Robin.Jones@rmh.nhs.uk

**Keywords:** angiosarcoma, angiogenesis, chemotherapy, immunotherapy, molecular biology, radiation-associated sarcoma, soft tissue sarcomas

## Abstract

**Simple Summary:**

Angiosarcomas are a group of a rare vascular cancers transformed from endothelial cells that could occur in any body part. Most angiosarcomas have unknown aetiology but secondary angiosarcomas could occur after radiation exposure or chronic lymphedema. The optimal treatment for localized angiosarcoma is complete surgical resection but neoadjuvant therapy may be helpful for some patients. For advanced angiosarcoma, systemic treatment including chemotherapy, anti-angiogenic therapy, and immunotherapies are options. We also review the molecular alterations that are associated with the pathogenesis of angiosarcoma, including c-Myc, TP53, vascular endothelial growth factor receptors, and others. This clinical management and molecular biology review will provide both clinicians and researchers of angiosarcoma with insights to help patients and move the field forward.

**Abstract:**

Angiosarcomas comprise less than 3% of all soft tissue sarcomas but have a poor prognosis. Most angiosarcomas occur without obvious risk factors but secondary angiosarcoma could arise after radiotherapy or chronic lymphedema. Surgery remains the standard treatment for localized angiosarcoma but neoadjuvant systemic treatment may improve the curability. For advanced angiosarcoma, anthracyclines and taxanes are the main chemotherapy options. Anti-angiogenic agents have a substantial role but the failure of a randomized phase 3 trial of pazopanib with or without an anti-endoglin antibody brings a challenge to future trials in angiosarcomas. Immune checkpoint inhibitors as single agents or in combination with oncolytic virus may play an important role but the optimal duration remains to be investigated. We also report the current understanding of the molecular pathways involved in angiosarcoma pathogenesis including MYC amplification, activation of angiogenic pathways and different molecular alterations that are associated with angiosarcomas of different aetiology. The success of the patient-partnered Angiosarcoma Project (ASCProject) has provided not only detailed insights into the molecular features of angiosarcomas of different origins but also offers a template for future fruitful collaborations between patients, physicians, and researchers. Lastly, we provide our perspective of future developments in optimizing the clinical management of angiosarcomas.

## 1. Introduction

Angiosarcomas (AS) are a heterogeneous group of rare mesenchymal tumours transformed from vascular endothelial cells, comprising less than 3% of soft tissue sarcomas and with an incidence rate of 2 to 3.5 per million [1]. They can occur in any anatomical location but more commonly occur in the head and neck and scalp regions, extremities, breast, heart and great vessels, and visceral organs such as the liver and spleen [1]. Secondary AS commonly arise after radiation exposure or chronic lymphedema. Among all radiation-associated sarcomas, AS is probably the most common histology [2,3]. The age at which AS occurs is diverse and mostly depends on the primary site and cause. Whereas cutaneous scalp AS tends to occur in the elderly (68.7% of patients were diagnosed at age older than 70) [4]. AS of the heart tend to occur in younger patients (67.1% were diagnosed at age younger than 54) [5]. Primary breast AS also occur in younger patients (median age 35 years) compared to secondary radiation-associated breast AS (median age 70 years) [6]. AS is an aggressive disease with 5-year survival of about 35% [1]. Even for localized AS with optimal surgery, the 5-year survival is less than 60% [1]. Chemotherapy or anti-angiogenic agents may be helpful, but resistance occurs quickly. The current understanding of the molecular features of AS has provided a deeper understanding of these tumours and could help us develop a more personalized approach toward treatment.

Angiosarcoma of bone is very rare, accounting for less than 1% of all primary bone sarcomas. This is a very aggressive variant but data are limited to retrospective studies. In a multi-center study of 80 patients with median follow-up of 31 months, the 5-year overall survival for patients with localized disease was 41% and for those with metastatic disease only 8% [7].

This review aims to provide a concise and up-to-date introduction to the aetiology, treatment, and molecular biology of AS and offers a perspective on the optimal strategy to move forward in the field of AS treatment and research.

## 2. Aetiology of Angiosarcomas

Most AS occurs spontaneously without identifiable risk factors, although cases of transformation from benign hemangioma or chronic hematoma have been reported [1]. Chronic lymphedema-associated AS, termed Stewart-Treves Syndrome, can occur in extremities after lymph node dissection or chronic infection such as filariasis [1]. Radiation exposure is an independent risk factor for secondary AS ; radiation-associated sarcoma typically occurs 5 to 10 years after radiation exposure [2], but recent reports have suggested that a shorter time interval may also be possible, especially with a history of concurrent chemoradiotherapy [8,9]. Any region that received radiotherapy is subject to secondary AS, but the most commonly reported areas included breast and head and neck regions. At present, risk factors or genetic factors associated with radiation-associated sarcomas remain elusive. Case reports of breast cancer patients with *BRCA1* or *BRCA2* mutations having post-radiation sarcoma have been reported [10], but a more extensive cohort study of *BRCA1* and *BRCA2* mutation carriers treated for breast cancer found no increased risk for radiation in-field secondary sarcoma after breast radiotherapy [11]. Other surrogate markers for radio-sensitivity, such as radiation-induced CD8 T-lymphocyte apoptosis (RILA), have also been implicated in identifying patients at risk for developing radiation-associated sarcoma [12]. Chemical exposure to vinyl chloride and thorium dioxide have been well-documented to be associated with increased risk of liver AS [13,14]. Workers with exposure to vinyl chloride had a standardized mortality ratio for liver cancer (60% were liver AS) of 3.59 (95% CI 2.84–4.46) [13].

## 3. Treatments for Localized Angiosarcoma

For localized AS, surgery remains the mainstay of curative treatment. Depending on the different primary anatomical location, different surgical approaches are necessary. For patients with extremity, truncal, and breast AS, complete resection with safe margins is the primary goal. Axillary lymph node dissection in breast AS remains controversial [15]. Angiosarcomas of the head and neck frequently involve a wide range of facial and scalp areas with no apparent demarcated margin. Along with the possible disfiguring complication of wide surgical resection, extensive surgery with clear margins are less likely to be achieved in this anatomical region. Angiosarcoma of the heart and great vessels are also challenging for surgery and often present with metastatic disease due to early haematogeneous spread. Some have proposed cardiac transplantation as a curative treatment for localized cardiac AS, but the long-term benefit remains to be confirmed [16,17]. Angiosarcomas of the great vessels such as the pulmonary artery often times masquerade as pulmonary embolism and undermines a proper plan for oncology surgery, limiting the opportunity for a complete resection [18]. The surgical management for secondary AS remains similar to primary AS, but the previous surgical history of the primary cancer and post-radiation effect may limit the surgery extension. The role of radiotherapy for AS as a sole treatment modality has not been investigated. Radiotherapy is mostly used as adjuvant therapy or reserved for patients unsuitable for surgery or systemic treatment. However, more than 50% of localized AS patients will develop recurrence or metastasis within five years [19,20]. Other treatment modalities are crucial to improve survival of localized AS.

### Neoadjuvant Systemic Treatment for Localized Angiosarcoma

The neoadjuvant systemic treatment strategy has the advantage to potentially down-size the primary tumour and also eradicate micrometastasis early. Those with tumour progression on neoadjuvant chemotherapy may be spared unnecessary surgery. Although no prospective randomized trials are available, retrospective studies have suggested that neoadjuvant systemic treatment is feasible with clinical benefit. Data from the European Organisation for Research and Treatment of Cancer (EORTC) institutions with 59 AS patients receiving neoadjuvant systemic treatment demonstrated that various systemic treatments, including chemotherapy and anti-angiogenic agents, have anti-tumour activity and could be maintained until maximum benefit; 10% of patients who received neoadjuvant treatment had disease progression before surgical intervention [21]. The median disease-free survival (DFS) and overall survival (OS) outcomes of the 86 AS patients, including the 59 patients who received neoadjuvant treatment, was 1.4 years and 4.9 years, respectively [21]. It is worth mentioning that 32% of the patients in this group had breast AS, and 28% were radiation-induced sarcomas.

Neoadjuvant chemotherapy in primary cardiac sarcoma may also provide benefit, even in patients with metastatic disease. In a retrospective study, Abu Saleh et al. showed that for right-side cardiac sarcomas (68% were AS), regardless of the metastatic status, patients treated with neoadjuvant chemotherapy had higher R0 resection rate (47 vs. 33%) and numerically better OS (20 vs. 9.5 months, *p* = 0.417) [22]. The ability of systemic therapy to reverse the surgical conundrum in AS was demonstrated in another prospective single-arm study of Oraxol—a combination drug of oral paclitaxel and a novel oral P-glycoprotein inhibitor, HM30181A. Among 18 locally advanced and inoperable but non-metastatic cutaneous AS patients, 5 (28%) inoperable patients become operable and received surgical resection. Although the primary outcome of the trial was not surgery conversion rate and long-term follow-up of the local control in these patients are awaited, these results suggest that neoadjuvant treatment could be considered for AS patients with localized or locally advanced disease [23]. Overall, neoadjuvant systemic treatment may be considered in AS on an individual basis, but currently there are no randomized data to confirm the benefit of this approach [24].

## 4. Advanced Angiosarcoma

The treatment for advanced AS typically includes systemic chemotherapy, anti-angiogenic agents, and recently novel agents such as immune checkpoint inhibitors (ICI). Because of the rarity of AS, few prospective clinical trials have been designed specifically for advanced AS. The ANGIOTAX and TAPPAS studies are two prospective clinical trials specially designed for AS patients; with the former study confirmed the role of paclitaxel in AS patients [25], and the latter showed no benefit for the anti-endoglin antibody in combination with pazopanib compared to pazopanib alone [26].

### 4.1. Chemotherapy in the Treatment for Advanced Angiosarcoma

#### 4.1.1. Anthracycline-Based Regimens

Understanding of the efficacy of anthracycline-based regimen in AS has mostly been generated from databases or clinical trials pooling patient outcomes for all types of soft tissue sarcomas. Generally speaking, anthracycline schedules have similar efficacy in AS compared to other non-angiosarcoma sarcoma histologies [27]. Within the 11 prospective EORTC clinical trials, the objective response rate (ORR), median progression-free survival (PFS), and median OS for the 108 advanced AS patients was 25%, 4.9 months, and 9.9 months, respectively [27]. Liposomal doxorubicin is a formulation of doxorubicin encapsulated by nanoparticle liposomes and has less cardiac toxicity than conventional doxorubicin [28]. However, inconsistent outcomes in treating soft tissue sarcomas with liposomal doxorubicin have resulted in its infrequent use except for Kaposi sarcoma [29,30,31]. Mechanistically, the enhanced permeability and retention effects of liposomes may partially explain the efficacy of liposomal doxorubicin in highly fenestrated vascular sarcomas such as Kaposi sarcoma [32]. Although prospective studies are lacking, retrospective studies have suggested that the liposomal doxorubicin may have similar efficacy for AS than single-agent paclitaxel. In a retrospective study by D’Angelo et al. [33], 31 AS patients treated with liposomal doxorubicin had an ORR of 35% and a median time-to-treatment failure of 5 months. In another retrospective study from the Asia Sarcoma Consortium, the median PFS of liposomal doxorubicin in first-line advanced AS was 2.8 months. However, the efficacy in terms of PFS was not significantly different from advanced AS patients treated with single-agent paclitaxel [34].

#### 4.1.2. Anti-Microtubule Agent-Based Regimen

Soft tissue sarcomas are generally insensitive to single-agent anti-microtubule agent therapies [35]. However, various studies have confirmed that AS is sensitive to single-agent paclitaxel. Retrospective studies have demonstrated that single-agent paclitaxel has comparable efficacy to anthracycline-based regimens in AS [36]. In the prospective ANGIOTAX study by Penel et al. [25], advanced AS patients treated with weekly paclitaxel had an 18% ORR and a median time-to-progression of 4 months. Interestingly, the efficacy of paclitaxel was not affected whether paclitaxel was given before or after the failure of an anthracycline [25]. Another anti-microtubule chemotherapeutic agent, eribulin, has also shown preliminary efficacy in cutaneous AS. In a prospective study by Fujisawa et al. [37] that was executed in Japan, 25 cutaneous AS patients resistant to paclitaxel received eribulin 1.4 mg/m^2^ D1 and D8 in a 21-day cycle. The ORR was 24%, and the OS and PFS rate at six months, estimated by the Kaplan-Meier method, was 67% and 24%, respectively. Lastly, there are also case reports of AS patients responding to anti-microtubule vinca alkaloids such as vinorelbine [38] or vinblastine [39].

#### 4.1.3. Other Chemotherapeutic Agents

Gemcitabine, a pyrimidine nucleoside prodrug, is also an alternative for AS treatment. In a retrospective review from the Italian Rare Cancer group, 25 AS patients were treated with single agent gemcitabine. The ORR was 68% and 6 out of 8 radiation-associated AS had tumour response. The median PFS and OS were 7 and 17 months, respectively [40]. Gemcitabine in combination with docetaxel is also commonly used in combination for soft tissue sarcomas. In a clinical trial of bevacizumab (a monoclonal anti-VEGF antibody), gemcitabine plus docetaxel, 3 out of 5 AS patients had a partial response [41].

### 4.2. Targeting the Anti-Angiogenic Pathway in Advanced Angiosarcoma

The growth of endothelial cells requires vascular endothelial growth factor (VEGF) and VEGF-receptor (VEFGR) axis activation. In AS, molecules associated with activation of angiogenesis often manifest through genetic mutations (KDR(VEGFR-2), PLCG1, PTPRB) [42,43] or amplification (FLT4(VEGFR-3)) [43]. Later sections of this Review will discuss angiogenic pathway activation and involvement in AS. In this section, we will focus on the outcomes of anti-angiogenic therapy in AS. Monoclonal anti-VEGF antibody bevacizumab did not show strong evidence of activity in AS. As a single agent, the ORR of bevacizumab in AS was 9% (2/23) [44]. In combination with paclitaxel, the addition of bevacizumab did not improve either ORR or median PFS outcome in AS [45]. The efficacy of various multi-targeted anti-angiogenesis tyrosine kinase inhibitors (TKI) has been explored in AS. Sorafenib, a TKI targeting VEGFR-2 and Raf, was associated with an ORR ranging from 15 to 23% and a median PFS of 3 months [46,47]. Pazopanib, a TKI that targets VEGFR 1, 2, and 3 as well as platelet-derived growth factor receptor (PDGFR)-alpha and beta and is currently a second or later-line treatment option of advanced STS [48], was shown to have an ORR of 20% in AS in a retrospective analysis of patients treated at EORTC centers [49]. The primary location (cutaneous vs. non-cutaneous) and cause (RT vs. non-RT associated) was not influential in determining the ORR of pazopanib in AS [49]. Other anti-angiogenic TKIs such as sunitinib and axitinib have occasionally reported having AS responders [50]. The angiopoietin-Tie axis is another essential pathway associated with vessel proliferation and remodeling, and the overexpression of TIE2, but not TIE1, was associated with worse outcome in AS [51]. However, in a phase I/II study testing anti-TIE1/TIE2 antibody trebananib, there were no responders out of 16 AS patients [52]. Overall, anti-angiogenic agent studies in AS patients showed that a proportion of AS depends on VEGF/VEGR axis to proliferate. However, the transient disease control suggests that other molecular pathways are pertinent in controlling AS patients’ outcomes. Durable responses were observed in AS patients treated in the Phase 2 trial of the FGFR/ VEGFR inhibitor brivanib [53].

#### The Rise and Fall of Anti-Endoglin Antibody in Angiosarcoma

Endoglin is crucial in directing endothelial cell proliferation and highly expressed in proliferating vessels in the tumour microenvironment. Furthermore, endoglin expression is commonly upregulated under hypoxic conditions, which can occur after angiogenesis inhibition [54]. In addition to the growth proliferating mechanism on endothelial cells, endoglin also plays a significant role in modulating mesenchymal cells [55]. Hence, targeting endoglin was well-positioned for soft tissue sarcomas, where both the cancer cells and the supporting vessels would be vulnerable to endoglin inhibition. TRC105 is an anti-endoglin antibody which was evaluated in a phase Ib/II trial of 81 sarcoma patients. Patients treated with a combination of pazopanib and TRC105 had a median PFS of 4.14 month. However, in the nine AS patients, the median PFS was 11.1 months, and two cutaneous AS patients had a complete response [56]. The promising outcome of the pazopanib and TRC105 combination in AS pivoted the development of TRC105 to AS in a confirmatory randomized phase III study—TAPPAS [57]. To accommodate the scarcity and heterogeneity of AS, TAPPAS employed an adaptive design that could adjust to different models of outcome after the first interim analysis. The clinical trial could adjust to one of the four models including unfavorable zone, enrichment zone (for cutaneous AS), promising zone, or favorable zone based on the conditional power of the full population and cutaneous subgroup after enrollment of 120 patients with 60 PFS events [57]. In 2019, after the accrual of 128 patients, the interim analysis recommended that the combination arm of pazopanib and TRC105 is likely to be futile in improving efficacy compared with single-agent pazopanib in AS, and TAPPAS was terminated early [26]. The median PFS and OS for the combination and pazopanib-only arm were 4.2 vs. 4.3 months, and not-reached vs. 8.0 months respectively. Interestingly, around 5–10% of AS patients were long-term survivors who had the disease under control for more than one year [26]. The disappointing outcomes of the TAPPAS study pointed out the heterogeneous nature of AS between cutaneous and visceral AS and the necessity to search for biomarkers to better understand the molecular signals in AS.

### 4.3. Immunotherapy in Angiosarcomas

The immune microenvironment of AS is less well understood, but AS from different primary locations may have different characteristics. For example, programmed death-ligand 1 (PD-L1) expression on tumour cells, which is considered a surrogacy of resistance to host immune activation, may be different in AS from different locations. The PD-L1 expression in cutaneous AS is around 40%, while primary breast AS tends to have lower PD-L1 expression than cutaneous AS [58,59,60]. Immune cells such as CD8^+^ and CD4^+^ T cells have been found occasionally in the tumour microenvironment of AS, but the impact of tumour-infiltrating T cells on survival has been inconsistent [59,60,61]. Most of the CD4^+^ or CD8^+^ T cells are memory T cells, as analyzed by flow cytometry [62]. The role of tumour-associated macrophages, which have an essential role in determining the tumour microenvironment for many types of sarcoma, has not been as well investigated in AS [63].

The success of immune checkpoint inhibitors (ICI) has revolutionized the treatment of many cancers. However, the outcomes of ICI in soft tissue sarcoma are not as straightforward. In the SARC028 clinical trial, single-agent pembrolizumab, a PD-1 antibody, had activity in 18% (7/40) of STS patients but was limited to specific histologies such as undifferentiated pleomorphic sarcoma and dedifferentiated liposarcoma [64]. However, the trial did not include AS patients. In the Alliance A091401 randomized, non-comparative trial, advanced sarcoma patients were randomized to either nivolumab or nivolumab plus ipilimumab combination. Two AS patients were randomized to the nivolumab plus ipilimumab arm, and one patient had a partial response by RECIST 1.1 with a duration of response of 6 months [65]. Cases of AS responding to either anti-PD-1/PD-L1 antibody or/and anti-CTLA-4 antibody have also been reported [62]. It is worth mentioning that not all AS responders to immunotherapy are long-term responders, with about half of the responses lasting only 3 to 6 months [62]. The combination of anti-angiogenic agents and ICI has also generated substantial interest and has been investigated in soft tissue sarcomas. However, the efficacy of these combinations in AS is still uncertain. The only AS patient in the axitinib and pembrolizumab combination trial had progressive disease at week 12 post-treatment [66]. One in two AS patients enrolled in the sunitinib plus nivolumab clinical trial had a partial response [26].

#### Combination of Viral Therapy and Immune Checkpoint Inhibitor

Tamilogene laherparepvec (T-VEC) is an oncolytic virus that demonstrated clinical efficacy in treating cutaneous melanoma [67]. After intra-tumoural injection of the virus, the virus replicates inside the tumour cells, kills the cancer cells, and releases tumour antigens. Combining an oncolytic virus with an anti-PD-1 antibody may further boost the immune response of the host to eradicate the tumour. In a phase II study of the combination of T-VEC and pembrolizumab in advanced soft tissue sarcomas, 7 out of 20 (35%) patients had an objective response rate by RECIST 1.1 [68]. Two of the three AS patients in the study had a partial response, with one of the AS responders being refractory to prior pembrolizumab single agent treatment. Although both AS patients completed 52 weeks of T-VEC plus pembrolizumab as per protocol, the investigators observed tumour progression after 1 to 4 months of treatment discontinuation. The disease was under control again after re-challenge of both patients with the combination [68]. Responding patients with histologies other than AS maintained disease control after completion and discontinuation of treatment. Overall, the optimal duration of immunotherapy for AS patients remains undetermined, and resistance may develop soon after the withdrawal of successful treatment. A summary of clinical trials investigating systemic therapies in angiosarcomas are listed in Table 1.

## 5. Molecular Biology and Translational Research in Angiosarcoma

The key molecular features of AS that are discussed herein are summarized in Table 2.

### 5.1. MYC Amplification

The *MYC* proto-oncogene is a transcription factor that regulates approximately 15% of all genes in the genome [110]. With such a high percentage of the genome under the control of MYC, it is unsurprising that this protein has a central role in tumorigenesis across many cancer types [111]. Aberrant MYC activity and overexpression leads to genome-wide dysregulation driving classic cancer hallmarks of proliferative signaling, altered cellular energetics and angiogenesis [112,113,114,115,116].

The presence of pathological *MYC* in AS was first reported through array comparative genomic hybridization (aCGH) and fluorescence in situ hybridization (FISH) studies [69,70,71]. aCGH analysis of 14 primary AS, 7 secondary radiation-associated AS, and 1 secondary lymphedema-associated AS revealed 16 recurrent alterations exclusive to secondary AS cases [70]. Detected in 57% of secondary AS cases, the most frequent alteration was amplification of the *MYC*-containing 8q24.12 chromosomal region.

FISH in an expanded cohort of 28 primary AS, 31 radiation-associated AS, and 2 lymphedema-associated AS cases detected *MYC* amplification in 55% of secondary AS, but not in primary AS; indicating *MYC* amplification is exclusive to secondary AS, and detectable irrespective of aetiology. Thus, although primary AS and secondary AS are morphologically indistinguishable, it is evident from this study that they possess genetic differences.

The amplification of *MYC* in other vascular lesions, such as atypical vascular lesions (ATVs), has also been assessed. Benign ATVs develop post-radiotherapy and share histological characteristics with the highly malignant radiation-associated AS, thus obtaining a correct ATV or radiation-associated AS diagnosis is challenging. FISH was employed to assess *MYC* amplification in 12 cases with slight vascular proliferation post-radiotherapy, 16 ATV cases, 22 radiation-associated AS cases, and 8 primary AS cases [71]. Strikingly, *MYC* amplification was detected exclusively in all radiation-associated AS cases and not in any primary AS cases, ATV cases, or cases showing slight vascular proliferation. This study extended previous observations, reporting *MYC* as being able to discriminate for secondary AS versus benign vascular lesions and primary AS. Subsequent studies have reproduced these results and highlights the potential utility of this protein in aiding the diagnosis of secondary AS [69,71,72].

However, despite numerous early reports documenting *MYC* amplification as exclusive to secondary AS, further studies with increased numbers of primary AS cases have shown contrary results. Three studies documented the first evidence of *MYC* amplification in primary AS [73,74,75]. As a result, a larger study to assess 38 archival primary AS cases was initiated [76]. Overexpression of the MYC protein, as measured by immunohistochemistry (IHC), was detectable in 24% of the primary AS cases. However, *MYC* amplification as detected by FISH analysis was reported in only 33% of the IHC-positive cases. Protein expression of MYC in the absence of gene amplification suggests alternative mechanisms of gene regulation are present within AS, and indeed, chromosome 8 copy number gains without *MYC* amplification were identified within this study. Another study also employed FISH to analyse a set of 10 radiation-associated AS, 1 lymphedema-associated AS, and 6 primary AS cases. This similarly identified *MYC* amplification as exclusive to 5 radiation-associated AS and 1 lymphedema-associated AS, and noted MYC overexpression by IHC in the absence of *MYC* amplification in 1 primary AS case [77]. Separately, FISH analysis of 32 radiation-associated AS and 15 primary AS cases reported *MYC* amplification in all radiation-associated AS and only a single primary AS [78]. Therefore, molecular alterations in *MYC* are also present in a subset of primary AS cases but at a much lower frequency compared to secondary AS.

The clinical consequence and downstream biology of molecular alterations in *MYC* in AS is largely unknown. However, there is some evidence to suggest a role for *MYC* in promoting the angiogenic phenotype [74]. MicroRNA (miRNA) sequencing of 8 *MYC*-amplified AS and 8 AS lacking *MYC* amplification revealed 43 significantly differentiated miRNAs. The most strongly upregulated miRNAs in the *MYC*-amplified cases were from the miR-17-92 cluster. The miR-17-92 cluster has previously been reported to amplify tumour angiogenesis in in vitro culture and mouse models, by repressing expression of anti-angiogenic proteins including thrombospondin-1 (THBS1) and connective tissue growth factor (CTGF) [79,80]. Moreover, quantitative assessment of THBS1 and CTGF mRNA levels found that these genes were downregulated in 13 *MYC*-amplified AS versus 12 AS lacking *MYC* amplification. This supports the hypothesis that MYC alterations promote angiogenesis in AS, however, as alterations are not found as ubiquitously across AS, it is evident that this is not an essential event for AS tumourigenesis, and the genetic basis for the disease is complex and diverse.

### 5.2. TP53 Alterations

The tumour suppressor *TP53* is one of the most commonly altered genes across sarcoma [117]. *TP53* alterations have a particularly high prevalence within leiomyosarcoma (80%) and pleomorphic sarcomas (60–70%) [81,118,119]. In AS, *TP53* mutations have been documented in a range of frequencies between 4% and 52% [42,81,82,83,84,85,86,87].

Although reports vary on exact incidence, it does appear that *TP53* mutations are less common in AS than in other sarcoma types. Evidence supporting a role for TP53 in AS tumorigenesis has been provided by studies involving transgenic models of disease. Zebrafish homozygous for tp53 deletion spontaneously develop tumours consistent with AS histology, as do mice with germline Trp53 deletion [88,90,91]. Furthermore, homozygous deletion of Trp53 in cadherin 5-expressing endothelial cells in mice also results in AS tumour development [89]. Interestingly, expression of a gain of function p53 mutant (R172H) in mouse endothelial cells does not lead to AS, but instead triggers lymphoma development [89]. In contrast, expression of the R172H mutant within pericytes results in 75% of mice developing AS, illustrating the importance of the cell of origin in defining AS tumorigenesis [89]. The development of AS in animal models through genetic manipulation of TP53 provides direct evidence that this tumour suppressor is important for this disease. Further mechanistic studies of TP53 dysfunction in the transgenic models discussed will facilitate elucidation of the role of this tumour suppressor in AS.

### 5.3. Alterations in Angiogenic Signaling Pathways

The vascular endothelial growth factor (VEGF) and VEGF receptor (VEGFR) family of proteins that control angiogenesis are found to be frequently altered in AS [117,120]. IHC analysis of 34 AS samples reported a high prevalence of VEGF/VEGFR expression; detecting VEGF-A, VEGFR-1, VEGFR-2, and VEGFR-3 in 94%, 94%, 65%, and 79% of cases, respectively [96]. Survival analysis demonstrated that patients with low VEGFR-2 expression had a significantly poorer OS versus those with high levels of VEGFR-2 (Hazard ratio = 5.16; 95% CI, 1.40–19.04 *p* = 0.014), however, the biological basis for this differential survival outcome is unclear. In addition to protein expression, somatic mutations in *VEGFR*-*2* have been identified in 0–33% of AS, with one study reporting a particularly high prevalence in 70% of breast primary AS [42,43,93,94,95]. *VEGFR*-*2* mutations occurred throughout the gene, including in the transmembrane, extracellular and kinase domains. However, 68% of all the documented mutations corresponded to T771 within the transmembrane domain.

Another documented *VEGFR* alteration is the amplification of VEGFR-*3,* often co-occurring alongside *MYC* amplification. Co-amplification has been detected by one study in 5 out of 28 secondary AS cases, and by another in 5 out of 20 secondary AS [74,92]. However, the exact prevalence and functional impact of this co-amplification event is unclear. Beyond the VEGF/VEGFR family itself, other angiogenic receptor tyrosine kinases (RTKs) and their ligands have also been shown to be dysregulated in AS. This includes the tyrosine kinase with immunoglobulin like and epidermal growth factor homology domains 1/2 (Tie1/2) and angiopoietin (Ang) ligands. Whilst VEGF signaling is crucial to initial vascularisation, Tie signaling is vital for long-term vascular maintenance [97]. Downstream of the Tie/Ang system are many other signaling pathways, including the focal adhesion kinase (FAK), mitogen-activated protein kinase (MAPK), and protein kinase B (PKB)/phosphoinositide-3-kinase (PI3K)/mammalian target of rapamycin (mTOR) axes. Therefore, overexpression or activation of Ang/Tie components is likely to trigger a cascade of increased signaling activity throughout these pathways, all of which are frequently implicated in tumorigenesis across cancer types [97,98,99,100,101]. In one study, moderate to strong expression of Tie-1, Tie-2, Ang-1, and Ang-2 has been reported by IHC in 68%, 80%, 86%, and 42% of AS cases respectively (*n* = 51) [51]. Furthermore, higher expression of Ang-1 was associated with a significantly improved OS; with no/weak Ang-1 expressing cases having a median OS of 5 months versus moderate/strong Ang-1 expressing cases of 47 months (*p* = 0.0049). The biological rationale for higher Ang-1 expression conferring improved OS is unclear. It is unlikely that in AS, higher Ang-1 expression is triggering enhanced signaling through the downstream FAK, MAPK, and PKB/PI3K/mTOR pathways, as excess activity in these pathways is well documented to enhance tumour progression [97,98,99,100,101].

Both the VEGFR and Tie signaling systems are under dynamic regulation, including by receptor-type tyrosine-protein phosphatase beta (PTPRB), which restricts VEGFR-2 and Tie-2 activity through dephosphorylation [102,103]. In a study by Behjati et al. [42], PTPRB mutations were identified in 10 out of 39 secondary AS cases, the majority being nonsense mutations occurring prior to or within the tyrosine phosphatase domain, resulting in loss of function of the protein. Implementation of a statistical maximum-likelihood model estimating selection pressures and pathogenic mutation rates showed these mutations to be statistically unlikely to present in AS by chance. Thus, loss of function mutations in *PTPRB* are hypothesised to be driver events in AS pathogenesis, leading to unmodulated Tie-2 and VEGFR-2 signaling. In addition to the identification of *PTPRB* mutations, the same study also found a recurrent R707Q missense mutation in phospholipase C gamma 1 (*PLCG1*) in 3 out of 34 cases [42]. This mutation has also been identified in subsequent studies; in 3 out of 10 primary cardiac cases profiled by targeted next generation sequencing, and in 8 of a larger 116-case study [43,95]. *PLCG1* encodes an enzyme responsible for membrane phospholipid hydrolysis, the products of which (inositol 1,4,5-trispohsphate (IP3) and diacylglycerol (DAG)) act as second messengers to stimulate several intracellular signaling pathways. One of the resultant pathways is angiogenic signaling, stimulated through VEGFR-mediated activation of PLCG1 [104,121]. Under typical conditions, the arginine 707 (R707) residue structurally supports the auto-inhibitory domain of PLCG1 [104]. In silico predictions illustrate that a glutamine substitution at this site will destabilise the domain, removing the auto-inhibitory capability of PLCG1 and resulting in a constitutively active form. This prediction is supported by in vitro evidence comparing human umbilical vein endothelial cells (HUVEC) ectopically expressing either PLCG1-wild-type or PLCG1-R707Q. Mutant-expressing cells showed abundant levels of inositol trisphosphate (IP3), a product of PLCG1-mediated cleavage, and had increased phosphorylation levels of the PLCG1-activated MAPK-pathway components [95]. Notably, although *PTPRB* mutations can co-present with either *PLCG1* or *VEGFR*-*2* mutations, as detected in 3 out of 10 *PTPRB* mutated cases by Beca et al., Huang et al. reported *PLCG1* and *VEGFR*-*2* mutations to be mutually exclusive in an analysis of 116 cases [42,43].

Dysregulated angiogenesis plays a central role in AS tumour development and progression. However, the exact mechanism for such dysregulation is highly varied across the AS patient population. As a result, although anti-angiogenic therapies may hold potential for the treatment and management of AS, a personalised approach may be needed to account for this inherent molecular heterogeneity.

### 5.4. Molecular Features of Angiosarcoma Associated with Aetiology

Molecular analyses of AS have been performed to differentiate between lesions of different aetiologies. Although alterations identified in MYC and the angiogenesis-related genes are often reported as having a slight preponderance for primary AS or secondary AS, the evidence is neither clear nor consistent. Several studies have undertaken comparative analysis of primary AS and secondary AS to identify molecular alterations that can provide a more robust discrimination between AS arising from different aetiologies.

In a study by Huang et al., targeted sequencing and FISH analysis of 120 AS cases (61% primary AS, 28% radiation-associated AS, 11% lymphedema-associated AS) reported primary AS-associated alterations in the capicua transcriptional repressor (*CIC*) gene [44]. *CIC* binds to gene promoters and enhancers to suppress target transcription [122,123]. Eight AS cases out of the 100 analysed by targeted sequencing carried unique missense mutations in exons 15 and 18–20 of *CIC*. Although the *CIC*-mutated cases included lesions with a range of different histological features, the group was highly enriched for younger patients, with those under 50 years old accounting for 7 cases. Furthermore, all but 1 of the *CIC*-mutated cases were primary AS. To a lesser extent, *CIC* gene rearrangements were also identified (*n* = 3). Notably, all 3 cases with *CIC*-rearranged cases were primary AS tumours showing epithelioid histology. The exact biological consequence of these *CIC* mutations and rearrangements is unknown; however, 2 *CIC*-rearranged cases showed upregulation of the *CIC*-target polyomavirus enhancer activator-3 (*PEA3*) family of transcription factors. It is therefore likely that CIC activity is suppressed or lost in a subset of primary AS cases.

Beyond the identification of single molecular alterations able to discriminate aetiology, Hadj-Mamou et al. [124] identified a gene expression signature capable of differentiating radiation-associated sarcomas from sporadic sarcomas. Gene ontology analysis revealed the signature was enriched for genes involved in mitochondrial processes and those associated with oxidative stress. To generate the signature, microarray analysis of a training cohort of 12 radiation-associated sarcomas and 12 sporadic sarcomas, each with 4 AS cases, 4 leiomyosarcomas and 4 osteosarcomas was performed with the identification of 135 genes that was able to discriminate between the two groups. This gene signature was then applied to the test cohort of 23 radiation-associated and 13 sporadic cases, each including 2 AS. As a result, 96% of radiation-associated sarcoma and 62% of sporadic sarcomas in the test cohort were correctly classified. This is still an active area of investigation as a more recent study using RNA sequencing reported indistinguishable transcriptomic profiles between 31 radiation-associated sarcomas and 53 sporadic sarcomas [125].

A follow-up study by the same group focused on AS and compared the transcriptome of 7 primary AS and 18 radiation-associated AS [73]. Applying the 135-gene signature to this cohort, 88% of case were assigned to the correct aetiology. Comparative analysis identified a further 42 genes with discriminatory value, including podoplanin (*PDPN*), and prospero homeobox 1 (*PROX*-*1*), which were upregulated in radiation-associated AS. PDPN is a marker of lymphatic endothelial cells, and PROX-1 regulates PDPN expression [126,127]. Therefore, this led to the hypothesis that radiation-associated AS initiates from lymphatic cells as opposed to vascular endothelia. Supporting this hypothesis, the study also reported *VEGFR*-*3* as amplified in 3 radiation-associated AS cases and VEGFR-3 is known to be involved in lymphangiogenesis [128].

Radiation-associated AS and primary AS are histologically distinct and some studies discussed herein do report key differences between the two aetiologies. Moving forward, there is a need to investigate whether findings associated with radiation-associated AS can be applied in the predictive setting to prospectively identify patients at high risk for radiation-associated AS development. Furthermore, molecular differences identified between radiation-associated AS and primary AS may also aid in the identification of new targeted therapies for this molecularly diverse disease.

### 5.5. The Future of Angiosarcoma Research: The Angiosarcoma Project

The majority of AS molecular studies have been limited in size due to the challenges associated with the collection of rare disease specimens. The AS project (ASCproject) is a recent patient-partnered initiative aiming to tackle this challenge by facilitating patient sharing of clinical data and samples across North America [105]. As part of this project, whole exome sequencing on tissue samples collected have been undertaken. To date, the project has enrolled nearly 350 patients, and the most recent project update in early 2020 describes findings from 227. The update included whole exome sequencing (WES) on 70 samples, 47 of which had a tumour purity >10% and so were used for analysis. These sequencing results support prior studies, documenting alterations in *MYC*, *VEGFR*-*3*, and *PLCG1*, and furthermore finds previously unreported mutations in *PIK3CA*, *GRIN2A* and *NOTCH2*. Of note, activating *PIK3CA* mutations were identified in 10 of 47 samples, 90% of which were breast primary AS lesions, raising the possibility that these patients may respond favourably to PI3Kα inhibitors [106]. A high tumour mutation burden (TMB) was also found in a subset of cases, specifically, in lesions of the head, neck, face and scalp. In addition, these samples showed an enrichment of the catalogue of somatic mutation in cancer (COSMIC) ultraviolet (UV) light exposure mutational signature. In-line with the high TMB and UV signature, 2 patients in this group had shown promising responses to off-label use of ICI. Indeed, high TMB has previously been reported as indicative for ICI response [107,108,109]. This highlights the potential for use of ICI in AS patients with high TMB and the UV signature. Without the centralised collection of data and specimens by the ASCproject, the geographical disparity of patient treatment sites would have made it challenging to make these discoveries, highlighting the importance of such collaborative patient-partnered initiatives for addressing gaps in knowledge and revealing new therapeutic targets in rare cancers.

## 6. Future Perspectives

Laboratory and clinical data indicate that AS is a very heterogeneous histological subtype. In addition, there is considerable variation in the management of localised disease as illustrated by a recent study by the EORTC Soft Tissue and Bone Sarcoma Group [27]. There has been much debate regarding the role of (neo)adjuvant chemotherapy in AS, but the design and conduct of a trial for this indication will be extremely challenging given the clinical and molecular variation within AS, but also the differing management strategies between sarcoma centers. However, it is hoped with a greater understanding of the molecular biology of AS it may be possible to perform such a trial.

Currently there are a number of systemic therapy options available to treat advanced AS. However, these treatments can result in a transient non-durable response, and it is clear that there is an unmet need for more effective chemotherapy schedules. The randomized Phase 3 trial of pazopanib with or without the anti-endoglin antibody in advanced AS showed no difference in progression-free or overall survival between the two arms [26]. However, a number of patients on the combination arm derived durable clinical benefit, suggesting a subgroup of patients with disease sensitive to this schedule. This reflects clinical observations of durable responses to specific systemic therapies in individual patients. An ongoing trial is evaluating an oral paclitaxel in cutaneous AS, with very promising provisional data both in terms of efficacy and durability of response [23]. This could prove to be a feasible therapy for patients with cutaneous AS, which tends to occur in the older population.

Furthermore, the work by Painter and colleagues suggests that cutaneous AS are sensitive to checkpoint inhibitors [105]. Future research should focus on identifying the molecular subgroups of AS, in order to identify patients most likely to benefit from specific chemotherapy schedules. This in turn should lead to the design of clinical trials for specific AS subgroups or stratified for such subgroups. Such trials will require international collaboration, which is clearly possible as the TAPPAS trial recruited over 120 patients in less than 2 years. Over the next decade we hope to see the development of more effective systemic therapy schedules for advanced AS and further evaluation of the role of systemic therapy in the localised setting.

## 7. Conclusions

The optimal clinical management of local disease is surgery, and neoadjuvant systemic therapy has the potential to improve curative surgery rate. For advanced AS, chemotherapy, anti-angiogenic agents are reasonable choices and immunotherapy should be considered for some AS patients with biomarker guidance. The revelation of abnormal angiogenic pathways, MYC, and CIC activation in AS has laid the foundation for future advancement in the understanding of pathogenesis and drug resistance of AS. Moving forward, researchers and physicians should partner with AS patients to improve patient outcomes in this heterogeneous and aggressive disease.

## Figures and Tables

**Table 1:** Clinical trials investigating systemic treatment activity in angiosarcoma.

Investigational Agent	Targeted Population	AS Specific	Single or Combo	*N*	Start Date	Expected Completion Date	Phase	NCT Identifier/EudraCT Number
Pazopanib	AS	Yes	Single	30	Nov 2011	Jan 2019	II	NCT01462630
Regorafenib	AS	Yes	Single	31	Jun 2014	Oct 2019	II	NCT02048722
Propranolol + metronomic cyclophosphamide	AS	Yes	Combo	24	Jan 2016	Not provided	I/II	2015-005177-21
Oraxol (oral paclitaxel + HM30181)	AS, cutaneous	Yes	Single	43	Dec 2018	Dec 2020	I/II	NCT03544567/2019-002085-13
Paclitaxel and avelumab	AS, metastatic	Yes	Combo	32	Jun 2018	Nov 2022	II	NCT03512834
T-VEC	AS, skin	Yes	Single	4	Arp 2019	May 2021	II	NCT03921073
Propranolol	AS, cutaneous and breast	Yes	Single	14	Dec 2019	Dec 2021	II	NCT04518124
Paclitaxel + radiotherapy	AS, cutaneous	Yes	Combo	19	May 2019	Dec 2021	I/II	NCT03921008
Paclitaxel + nivolumab Cabozantinib + nivolumab *	AS of skin, radiation-associated skin, and visceral	Yes	Combo	90	Sep 2020	Sep 2023	II	NCT04339738
AGN2034 + AGEN 1884	AS	Yes	Combo	55	Oct 2020	Oct 2021	II	NCT01042379
Doxorubicin + dexrazoxane	STS, including AS	No	Combo	73	Feb 2016	Oct 2023	II	NCT02584309
Nivolumab + ipilimumab	Rare cancers, including AS	No	Combo	818	Jan 2017	Aug 2021	II	NCT02834013
T-VEC + pembrolizumab	STS, including AS	No	Combo	60	Mar 2017	Mar 2021	II	NCT03069378
Sunitinib + nivolumab	STS and bone sarcoma, including AS	No	Combo	270	Mar 2017	Sep 2022	I/II	NCT03277924/2016-004040-10
Ribociclib and doxorubicin	STS, including AS	No	Combo	16	Mar 2017	Oct 2019	I	NCT03009201
L19TNF + doxorubicin vs. doxorubicin	STS, including AS	No	Combo	102	July 2017	Not provided	III	2016-003239-38
Durvalumab + tremelimumab	Sarcoma, including AS	No	Combo	62	Aug 2017	Aug 2020	II	NCT02815995
RP1 +/− pembrolizumab	Solid tumor, including AS	No	Combo	293	Aug 2017	Not provided	I/II	2016-004548-12
Durvalumab + trememlimumab vs. doxorubicin	STS, including AS	No	Combo	100	Oct 2017	Not provided	II	2016-004750-15
Eribulin	AS and EHE	No	Single	16	Jan 2018	May 2021	II	NCT03331250
Atezolizumab + radiotherapy	STS, including AS	No	Combo	69	Feb 2018	Not provided	II	2016-005019-42
SPM-011 + Boron Neutron Capture Therapy	AS and melanoma	No	Combo	9	Nov 2019	Dec 2021	NA	NCT04293289
Cobimetinib + atezolizumab	STS, including AS	No	Combo	80	Oct 2019	Not provided	I/II	2019-000987-80

(last accessed 2020/10/30 from https://www.clinicaltrials.gov and https://www.clinicaltrialsregister.eu/. Keywords: angiosarcoma; recruitment status included “not yet recruiting”, “recruiting”, and “active, not recruiting.” for ClinicalTrials.gov and “Ongoing” for EU Clinical Trial Register). AS: angiosarcoma, EHE: epithelioid hemangioendothelioma, STS: soft tissue sarcoma, T-VEC: tamilogene laherparepvec.* The cabozantinib plus nivolumab arm enrolls patients who are refractory to paclitaxel.

**Table 2:** Summary of the key molecular features of angiosarcoma, with the frequency of genomic alteration, and if known the biological and clinical consequences.

Molecular Feature	Genomic Alteration	Frequency	Biological Consequence	Clinical Consequence
MYC transcription factor	*MYC* amplification	50–100% secondary AS; 7–8% primary AS [69,70,71,72,73,74,75,76,77,78,79]	Hypothesised to promote the angiogenic phenotype through upregulation of miRNAs in the miR-17-92 cluster, leading to reduced expression of anti-angiogenic THBS1 and CTGF [79,80].	−
	MYC overexpression	17–24% primary AS [76,77]		−
TP53	*TP53* mutations	4–52% AS [42,81,82,83,84,85,86,87]	Exact biological consequence in AS unknown. Animal models with TP53 alterations go on to develop AS [88,89,90,91].	−
Vascular endothelial growth factor (VEGF) & VEGF receptor (VEGFR) signaling	*VEGFR-3* amplification (*MYC* amplification co-occurrence)	18–25% secondary AS [76,92]	Uncontrolled VEGF/VEGFR signaling leads to dysregulated angiogenic activity, however exact mechanism in AS remains unknown.	−
	*VEGFR-2* mutations (68% at T771)	0–33% AS; 70% breast primary AS [42,43,93,94,95]		−
	VEGF/VEGFR family overexpression	94% AS (VEGF-A); 94% AS (VEGFR-1); 65% AS (VEGFR-2); 79% AS (VEGFR-3) [96]		Potential prognostic value: lower VEGFR-2 expression associated with significantly poorer OS [96]
Tyrosine kinase with Ig like and EGF domains (Tie) and Angiopoietin (Ang) signaling	Tie/Ang system overexpression	68% AS (Tie-1); 80% AS (Tie-2); 86% AS (Ang-1); 42% AS (Ang-2) [51]	Exact biological consequence in AS unknown. Increased signaling activity through the Tie/Ang system is likely to trigger a cascade of increased activity through the downstream FAK, MAPK, PKB/PI3K/mTOR axes [97,98,99,100,101].	Potential prognostic value: lower Ang-1 expression associated with significantly poorer OS [51]
Receptor-type tyrosine-protein phosphatase beta (PTPRB)	*PTPRB* mutations	26% secondary AS [42]	Loss of function mutations are most commonly observed and are hypothesised to lead to unmodulated Tie-2 and VEGFR-2 signaling [102,103].	−
Phospholipase C gamma 1 (PLCG1)	*PLCG1* mutation (R707Q)	7–9% secondary AS; 30% cardiac primary AS [42,43,95]	Mutation results in constitutively active PLCG1 leading to increased MAPK pathway signaling [95,104].	−
Capicua transcriptional repressor (CIC)	*CIC* mutations	8% AS; enriched for young primary AS patients [43]	Exact biological consequence in AS unknown. CIC-rearrangements hypothesised to reduce CIC activity, leading to upregulation of CIC-target PEA3 family transcription factors [43].	−
	*CIC* re-arrangements	3% AS; enriched for primary AS cases with epithelioid histology [43]		−
Phosphatidylinositol-4,5-bisphosphate 3-kinase subunit alpha (PIK3CA)	*PIK3CA* mutations	21% AS; enriched for breast primary AS cases [105]	Exact biological consequence in AS unknown, however activating mutations were observed, and likely lead to increased signaling activity.	Potential treatment option: subset of patients may respond to PI3Kalpha inhibition [106]
Tumour mutation burden (TMB)	High TMB	21% AS; enriched for head, neck, face & scalp cases [105]	Exact biological consequence in AS unknown. High TMB present alongside COSMIC UV signature [105].	Potential treatment option: subset of patients may respond to immune checkpoint inhibition [105,107,108,109]
